# Laparoscopic Management of a 12-Week Pregnancy Loss in a Rudimentary Uterine Horn

**DOI:** 10.7759/cureus.61677

**Published:** 2024-06-04

**Authors:** Tara Srinivas, Gregory W Kirschen, Golsa M Yazdy

**Affiliations:** 1 Gynecology and Obstetrics, Johns Hopkins Hospital, Baltimore, USA

**Keywords:** early pregnancy loss, preterm birth, laparoscopic surgery, ectopic pregnancy, mullerian duct anomaly

## Abstract

Unicornuate uterus with rudimentary horn is a rare structural uterine anomaly resulting from incomplete Mullerian duct development and/or fusion. Pregnancy in rudimentary horn is an uncommon presentation of a Mullerian anomaly and may lead to substantial morbidity and mortality due to high risk of uterine rupture with intraabdominal hemorrhage. Medical and/or surgical management may be undertaken; however, currently, no treatment guidelines exist. We describe the management of a 12-week rudimentary horn pregnancy in a 25-year-old multiparous patient with a prior spontaneous preterm breech vaginal delivery and one spontaneous early term cephalic vaginal delivery in whom this congenital uterine condition was previously unknown. The rudimentary horn, nonviable pregnancy, and contiguous ipsilateral fallopian tube were excised laparoscopically without complication. Given the infrequency of rudimentary horn pregnancies and the high risk for obstetric complications, a high index of suspicion should be maintained. We emphasize that a history of preterm birth or malpresentation should raise suspicion for maternal Mullerian anomaly, and that a minimally invasive approach can be feasible for treatment of a rudimentary horn pregnancy.

## Introduction

A unicornuate uterus with a rudimentary horn is characterized by the American Fertility Society/American Society for Reproductive Medicine (ASRM) as a Mullerian anomaly that results from the incomplete development or fusion of one paramesonephric duct, leaving a uterus with only one fully developed fallopian tube. Incomplete atresia of the affected contralateral Mullerian duct results in a remnant of the rudimentary horn that may or may not communicate with the endometrial cavity [1].

During embryonic development, paired Mullerian ducts, composed of intermediate mesoderm, fuse laterally between the seventh and ninth weeks of gestation [2]. Failure of this fusion leads to various anomalies ranging from subtle changes in the shape and architecture of the uterus to the complete absence of the uterus, cervix, and upper vagina (Mullerian agenesis) [2]. The ASRM classifies Mullerian anomalies by anatomical presence, absence, or abnormal development of the uterus, cervix, and upper vagina [1]. When there is underdevelopment or abnormal fusion of the upper Mullerian ducts, a unicornuate uterus can result, with a spectrum of anomalies involving either a truncated uterine horn that communicates (via endometrial and myometrial bridge) with the dominant horn, a small atretic contralateral uterine horn that does not communicate, or the complete absence of the contralateral horn [1].

Unicornuate uterus with rudimentary horn occurs in approximately 0.17% of women (4% of women have a Mullerian anomaly, of which 5% are unicornuate uterus, of which 83% are accompanied by a rudimentary contralateral horn) [3] and is primarily considered a de novo congenital occurrence [4].

Approximately 75-90% of unicornuate uteri with a rudimentary horn are non-communicating [5], meaning there is no connection between the remnant paramesonephric duct and the endometrial cavity [1]. In such cases, pregnancy may be achieved by transperitoneal migration of the sperm or zygote [6]. Pregnancy in a rudimentary uterine horn is extremely rare (approximately 1:76,000 to 1:140,000 pregnancies) and morbid, with a 6% rate of neonatal survival and an 80% rate of uterine rupture by the end of the second trimester ​[6-8].

Here, we present a case of a 25-year-old patient who underwent emergent laparoscopic surgery for the management of suspected tubal ectopic pregnancy and was found to have a rudimentary horn pregnancy secondary to a congenital anomaly. The patient’s written informed consent was obtained for this case report. IRB approval was not required for this study per institutional policy.

## Case presentation

A 25-year-old, G3P1101, at 19 weeks and three days of gestation, presented to the emergency department with a one-day history of vaginal bleeding and abdominal cramping. The quantitative β human chorionic gonadotropin (β-hCG) level was 519.0 mUI/mL.

Prior obstetric history included one uncomplicated early-term vaginal delivery at 37 weeks and one preterm breech vaginal delivery at 34 weeks, resulting in neonatal death due to cardiac etiology. Both pregnancies had taken place at outside institutions. The patient had no known pertinent gynecological history.

**Figure 1:**
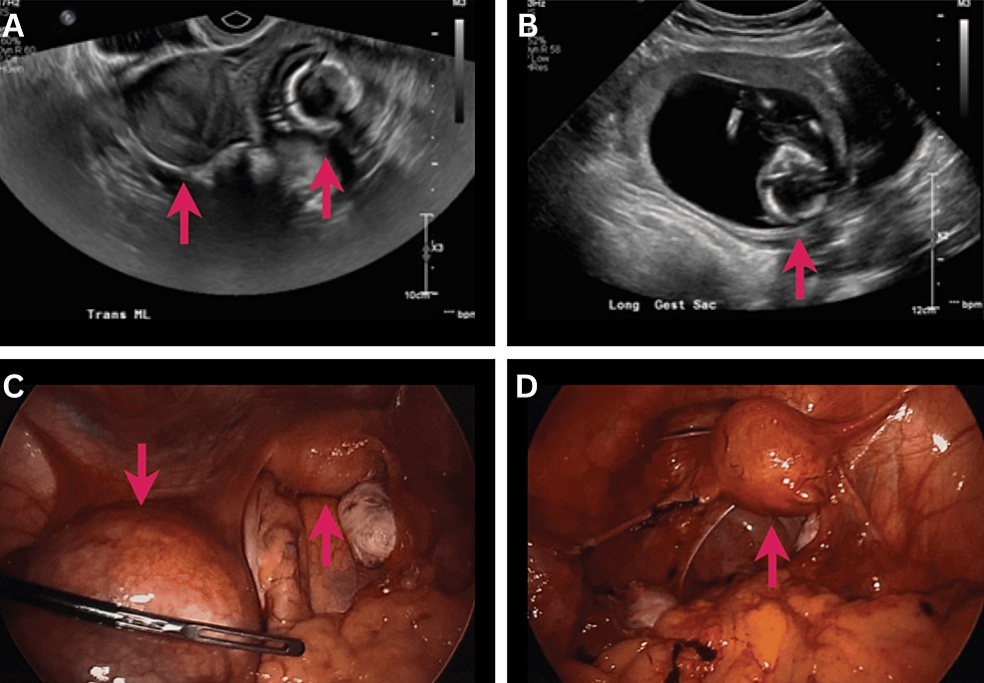
Ultrasound and intraoperative images demonstrating left rudimentary uterine horn pregnancy (A) Transvaginal ultrasound showing an empty dominant uterine horn (left red arrow) and a rudimentary horn containing pregnancy (right red arrow). (B) Transabdominal ultrasound showing fetal calvarium in the left adnexal region (red arrow). (C) Intraoperative visualization of the unicornuate uterus (right red arrow) with the rudimentary horn containing gestation (left red arrow, in contact with the Dorsey grasper) before excision. (D) Laparoscopic image of the remaining dominant horn (red arrow) after rudimentary horn excision.

Transabdominal and transvaginal (Figure 1A-1B) ultrasound impressions described an extrauterine pregnancy with a crown-rump length of 61.16 mm, corresponding to a gestational age of 12 weeks and four days in the region of the left adnexa without fetal cardiac activity. There was no evidence of hemorrhage or hematoma to suggest a ruptured tubal ectopic or uterine horn. Given the high concern for an ectopic pregnancy of advanced gestational age, surgical management was recommended.

In the operating room, general anesthesia was administered and diagnostic laparoscopy was performed given the concern for tubal ectopic pregnancy, which surprisingly revealed two horns: a dominant right uterus and a secondary left rudimentary uterine horn containing the gestation (Figure 1C). Laparoscopic resection of the left rudimentary uterine horn and left salpingectomy were performed with the LigaSure device (Medtronic, Minneapolis, MN, USA). The specimen was placed in an endocatch bag and morcellated through the umbilicus. Myometrium and fetal parts were confirmed upon incision of the rudimentary horn during morcellation. Post-excision, pelvic laparoscopy revealed normal bilateral ovaries, the dominant unicornuate uterus to the right of the mid-sagittal plane, and a normal right fallopian tube (Figure 1D).

The patient was admitted for overnight observation and was discharged in stable condition on postoperative day one. A follow-up renal sonogram was undertaken to assess for associated urological anomalies [2] and showed no evidence of urinary tract anomalies. The final pathology revealed portions of the uterus and fallopian tube with necrotic fetal parts and chorionic villi.

## Discussion

Rudimentary horn pregnancy is a rare occurrence in individuals with Mullerian anomalies. Recent trends show a decrease in mortality from 23% to 0.5% among these patients [9,10], possibly due to advancements in an increased frequency of pelvic imaging. It is important to detect Mullerian anomalies before pregnancy to avoid serious obstetric complications, including uterine rupture and preterm birth; however, only an estimated 14% of cases are diagnosed prior to clinical manifestations [11]. In the present case, transvaginal ultrasound raised suspicion for adnexal pathology, and a diagnosis of uterine anomaly was only made upon diagnostic laparoscopy.

Evidence suggests that the sensitivity of transvaginal ultrasound is indeed low (~25-30%) for the diagnosis of rudimentary horn before pregnancy [11,12]. Three-dimensional ultrasound and pelvic MRI may improve the detection of Mullerian anomalies, guide operative planning [11], and be helpful when there is a high index of clinical suspicion. Additionally, any patient with a history of one or more preterm births or malpresentations should raise suspicion for a possible underlying Mullerian anomaly [13]. Currently, there are no guidelines for the treatment of a rudimentary horn pregnancy. Among the case literature, excision of the pregnant horn and ipsilateral fallopian tube is commonly reported, with a few cases of successful management with methotrexate or a combined medical and surgical approach [11,14]. Treatment rationale does not appear to differ between communicating and non-communicating pathologies. In the present case, rapid surgical intervention was undertaken despite hemodynamic stability due to the risk of rupture and concern for a possible ectopic pregnancy. The patient was counseled postoperatively regarding the maintenance of fertility and the possibility of a future pregnancy [15].​ Based on thin peritoneal tissue between the two horns and no apparent disruption to the remnant cavity, we are confident that in the present case, the two horns were not communicating. On the other hand, in cases of communicating rudimentary horn excision involving disruption of the dominant horn cavity, postoperative counseling may include a discussion of cesarean sections for future pregnancies based on prior literature [14,16]. However, guidelines for this recommendation are lacking.

## Conclusions

This case highlights challenges and delays in the diagnosis of Mullerian anomalies, as well as a good outcome with a minimally invasive approach. Future studies should evaluate the long-term clinical and functional outcomes, including effects on fertility, of patients with communicating and non-communicating pathologies to further develop treatment recommendations.
